# Clinical Heterogeneity and Transitions of Obesity in Mexico. A Longitudinal Analysis of Multiple Representative National Surveys

**DOI:** 10.1210/clinem/dgaf158

**Published:** 2025-10-16

**Authors:** Adrian Soto-Mota, Rodrigo M. Carrillo-Larco, Edward Gregg, Rosalba Rojas Martínez, Majid Ezzati, Carlos Aguilar-Salinas

**Affiliations:** 1 Metabolic Diseases Research Unit, National Institute of Medical Science and Nutrition Salvador Zubiran, Mexico City, Mexico; 2 Tecnologico de Monterrey, School of Medicine, Mexico City, Mexico; 3 Hubert Department of Global Health, Rollins School of Public Health, Emory University, Georgia, United States; 4 MRC Center for Environment and Health, School of Public Health, Imperial College London, London, United Kingdom; 5 School of Population Health. Royal College of Surgeons in Ireland; 6 Instituto Nacional de Salud Pública, Mexico City, Mexico; 7 Regional Institute for Population Studies, University of Ghana, Accra, Ghana

**Keywords:** Obesity, Mexico, ENSANUT, Diabetes, Hypertension, Dyslipidemia, multi-morbidity

## Abstract

**Background:**

There is large variation in the individual risk of developing obesity-associated comorbidities. While obesity is highly prevalent in Mexico, data on the extent and heterogeneity of its associated co-morbidities is lacking. Hereby, we estimated the prevalence of different obesity-associated comorbidities, and how they have changed over 15 years.

**Methods:**

We gathered data from different editions of nationally representative health and nutrition surveys (ENSANUT) from 2006 to 2022. The prevalence of obesity and the coexistence with diabetes, dyslipidemia, hypertension, depression, and impaired mobility, which are outcomes used in the Edmonton Obesity Staging System (EOSS) which assesses three dimensions (medical, mental, and functional) across five incremental severity stages, by sex and age groups were estimated across all included surveys. Metabolically healthy obesity was defined as the absence of diabetes, dyslipidemia and hypertension.

**Results:**

20758 participants were analyzed. Mean BMI increased progressively at all ages from 30.2 to 31.0 across survey rounds. Depression and impaired mobility were highly prevalent even among metabolically healthy obese individuals. While most people with obesity had at least one detectable abnormality, there was large heterogeneity in the presented comorbidities. The most prevalent EOSS categories were stage 2 for the medical dimension (90.1%), and stage 1 for the functional and mental dimensions (75.1% and 62.9%, respectively). The prevalence of obesity-related comorbidities increased with age but was similar across all surveys. In both sexes, metabolically healthy obesity was less likely as age and BMI increased.

**Conclusion:**

The prevalence of obesity comorbidities has been stable over time in Mexico but increases with age. The rising prevalence of obesity and the ageing of the population will cause additional burdens to the population and the health system.

## Introduction

Obesity is highly prevalent and has increased worldwide^1^ and its direct association with many different clinical conditions has been established^2^. While it is generally recognized that obesity coexists with other independent risk factors for the same poor health outcomes, most reports have focused on its interaction with metabolic diseases or cardiovascular risk factors (i.e. hypertension and dyslipidemia) without having a multifaceted description of the burden caused by obesity in individuals and populations.^3^ On the other hand, it has also been recognized remarkable variation in the individual risk of developing obesity-associated comorbid diseases. Consequently, the impact of obesity on health and quality of life outcomes needs to be comprehensively assessed^4^. Some groups have proposed the stratification of the disease by sub-phenotypes, such as metabolically healthy (MHO) or unhealthy obesity (MUO). Controversy exists about the pathophysiology and clinical relevance of the MHO diagnosis. While many authors consider MHO as a stage of the natural history of obesity, others conceptualize it as a stable condition associated with a better prognosis^5^. Other authors have based the sub-phenotyping on psychological variables^6^ or even pathophysiologic mechanisms^7^. However, none of these approaches assesses the impact of obesity on other health-relevant but non-metabolic factors.

Mexico is among the countries with the highest prevalence of obesity. Major socioeconomic changes occurred between 1960 and year 2000 resulting in remarkable modifications in eating patterns and health habits^8^. Despite the recognition of obesity as a major driver of health costs and the implementation of public policies, the prevalence of obesity continues to rise^9^. In contrast, despite its strong association with obesity, not all comorbidities have increased its prevalence at the same rate. The available reports provide detailed information about the epidemiology of obesity in Mexico without considering its impact in non-metabolic dimensions^10–12^. While it has already been documented that the prevalence of obesity has been rising in Mexico from 25.1% in 2000 to almost 40% in recent years^12^, estimating the prevalence of obesity-related comorbidities and its trends could yield additional and useful insight for informing public policy, we sought to compare the prevalence of obesity and its associated comorbidities in Mexico between 2006 and 2022 using data from multiple editions of its representative National Nutrition and Health Survey (ENSANUT)^12–14.^

Additionally, we measured the prevalence of co-existing comorbidities using the clinically validated Edmonton Obesity Staging System (EOSS), which was developed in 2009 in line with the World Health Organization’s holistic definition of health to also consider functional and mental factors using a 5-stage scale that ranges from subclinical to end-stage severity^13,14,15^.

## Methods

### Ethical Approval

Every sampling round of ENSANUT was approved by the Ethics Committee of the Instituto Nacional de Salud Publica and collected written informed consent. Anonymized databases are available to the public (https://www.insp.mx/centros/evaluacion-y-encuestas/uisp/encuestoteca.html). As no new data was generated, no new ethical approval was requested for this study.

### Data

ENSANUT probabilistic survey that employs a stratified multi-stage sampling approach to ensure the collection of representative data on health and nutrition across Mexico. No participant is tested in more than one ENSANUT edition, the survey divides the population into various strata based on geographic and socioeconomic characteristics, ensuring comprehensive coverage of all significant subpopulations. Within each stratum, clusters are randomly selected, followed by systematic sampling of households within these clusters. This design is enhanced by proportional allocation, where larger strata contribute more to the sample, and the use of sampling weights to adjust for unequal probabilities of selection and non-response. Additionally, the survey allows oversampling of certain subpopulations to ensure robust data for these groups. The survey employs a variety of instruments, including household questionnaires and physical measurements, as detailed elsewhere^16^. The only additional inclusion criteria in this analysis were a BMI between 24.9 and 29.9 for the analyses on people with overweight or a BMI above 29.9 for the analyses on people with obesity.

### Variable Definitions

Overweight was defined as a BMI of 25 to <29.9 and obesity as a BMI of 30 kg/m^2^ or greater. ^13^ Within our working definitions, the EOSS medical dimension includes dyslipidemia, diabetes or hypertension while the mental dimension only considers depression and the functional dimension only considers mobility issues. Diabetes was considered present if the diagnosis was made by a health professional and/or HbA1c concentration was > 6.5% or fasting glucose levels were≥126 mg/dl. A systolic pressure >140 mmHg or a diastolic pressure >90 mmHg and/or the diagnosis was made by a health professional and or the use of medications were the diagnostic criteria for arterial hypertension. All blood pressure measurements were made by duplicate and by trained personnel. Dyslipidemia was defined as total cholesterol (CT) >200 mg/dL, and/or triglycerides >150mg/dL, and/or HDL<40mg/dL, and/or non-HDL > 130, and/or the use of lipid-lowering medications. Depression and Mobility issues were considered present following the working definitions of their respective screening questionnaires which require at least 2 positive answers (for more details see^17^).

Beyond the presence of comorbidities, EOSS considers their severity in a staging system. In the EOSS, stage 0 means the absence of abnormalities. Stage 1 means subclinical risk factors which we defined as having prediabetes or systolic pressure ≥ 120 mmHg but ≤ 140 or diastolic pressure ≥ 80 mmHg but ≤ 90 or only 1 depression symptom. Stage 2 means an established disease as described below, and Stage 3 or above (which is defined in the EOSS framework as severe disease or end-stage disease) was defined as fasting glucose ≥ 200 mg/dL or systolic pressure ≥ 160 mmHg or diastolic pressure ≥ 90 mmHg or non-HDL ≥ 190 mg/dL. Stage 3 and above levels were not available for the Mental and Functional Dimensions as no further severity data was available. Since the same patient frequently meets more than one dyslipidemia criterion, the frequency of each dyslipidemia sub-type was calculated as the proportion of patients meeting each criterion over the total of patients with dyslipidemia.

Additionally, the 2016 survey included serum liver disease markers. As reported before^18^, non-alcoholic fatty liver disease (NAFLD) was defined as having Fatty Liver Index (FLI) > 60. The FLI was calculated with the following formula (FLI = (e 0.953*loge (triglycerides) + 0.139*BMI + 0.718*loge (GGT) + 0.053*waist circumference - 15.745) / (1 + e 0.953*loge (triglycerides) + 0.139*BMI + 0.718*loge (GGT) + 0.053*waist circumference - 15.745) * 100)^19^. Similarly, the 2016 and 2018 ENSANUT editions had kidney function markers. We estimated the glomerular filtration rate using the R function *transplantr::ckd_epi*. Chronic kidney disease was defined as an estimated glomerular filtration rate < 30 mL/min/1.73m^2^. A cut-off of eGFR between 30 and 60 was used for defining Stage 1 cases for this particular outcome.

### Statistical Analysis

All datasets were cleaned according to a previously specified protocol for the NCD-RisC collaboration which is available at: https://github.com/NCD-RisC/Data-cleaning-protocol. Data management and statistical analyses were performed using R version 4.0.3.

All descriptive statistics accounted for the complex sampling method of ENSANUT^16^ using survey::svydesign. Unless otherwise stated, all descriptive analyses are reported as means, and standard deviation and prevalences are reported as percentages. To estimate the relevance of explaining the observed variability of age, sex, and BMI on the number of obesity-related comorbidities, we used multivariable linear models and compared their R^2^.

## Results

### Data cleaning results and population composition changes across surveys

After data cleaning, 20758 participants from 5 national surveys from 2006 to 2022 were eligible for analysis (Supplemental Figure 1)^19^. During this period, the Mexican population went through age composition and anthropometric changes with an overall trend towards an older population (see Supplemental Figure 2) ^19^ and the mean crude BMI increasing from 30.2 in 2006 to 31.0 in 2022 and from 29.9 to 30.9 among young adults (≤35 years old) (see Supplemental Figure 3) ^19^.

### The heterogeneity of the prevalence of obesity-related comorbidities

**Figure 1:**
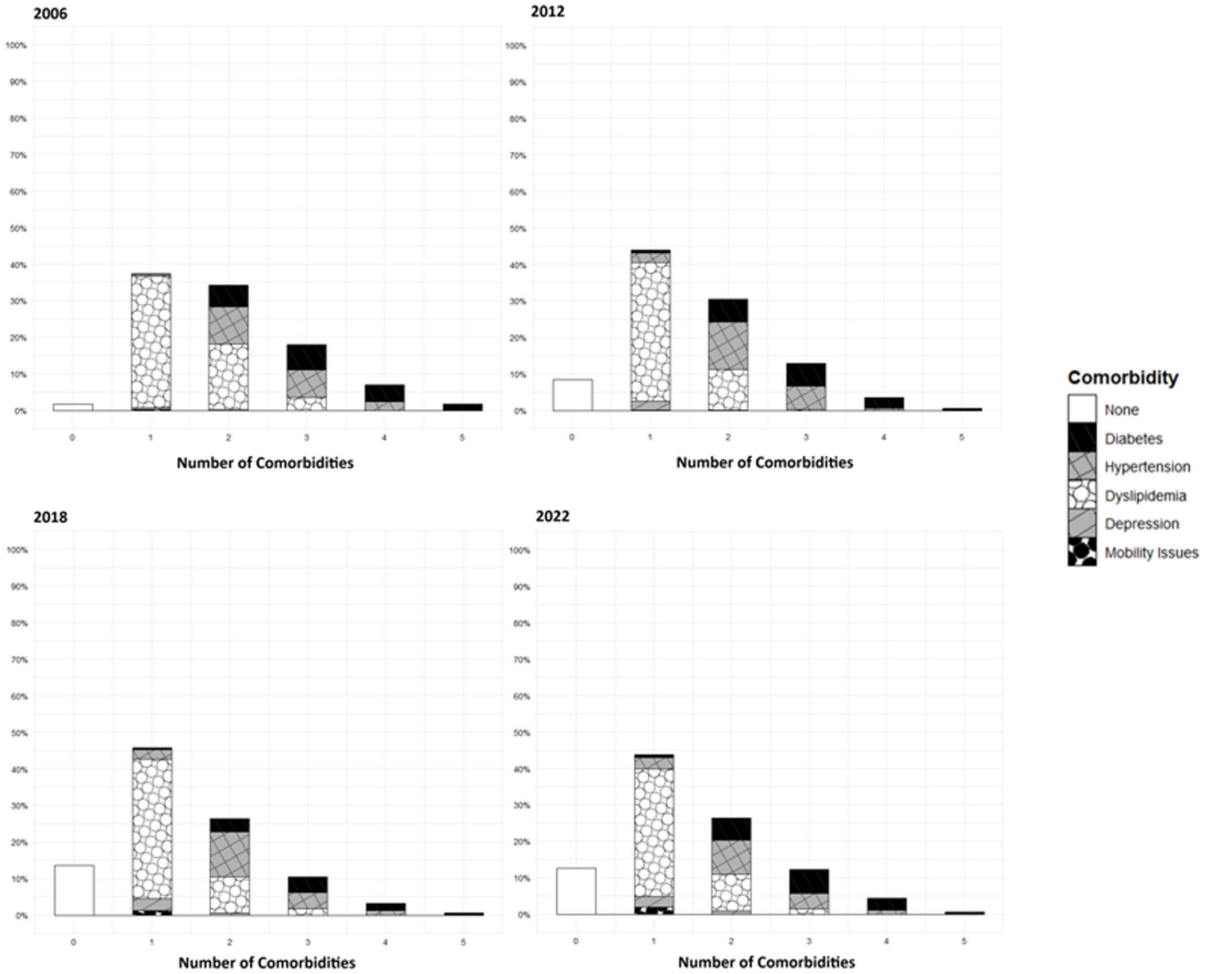
Type and Number of Obesity-Related Comorbidities Across Time. While the prevalence of each comorbidity was statistically different in every edition, they remained comparable in magnitude except for the prevalence without any comorbidities

In all surveys, most participants had at least one obesity-related comorbidity. Dyslipidemia was the most frequently observed obesity-related comorbidity. On the other hand, there was an increase in the prevalence of metabolically healthy obese participants in the two most recent ENSANUT editions (Figure 1). Elevated non-HDL (71%) cholesterol and hypertriglyceridemia (52%) were the most common dyslipidemia sub-types (see publicly available code).

**Figure 2:**
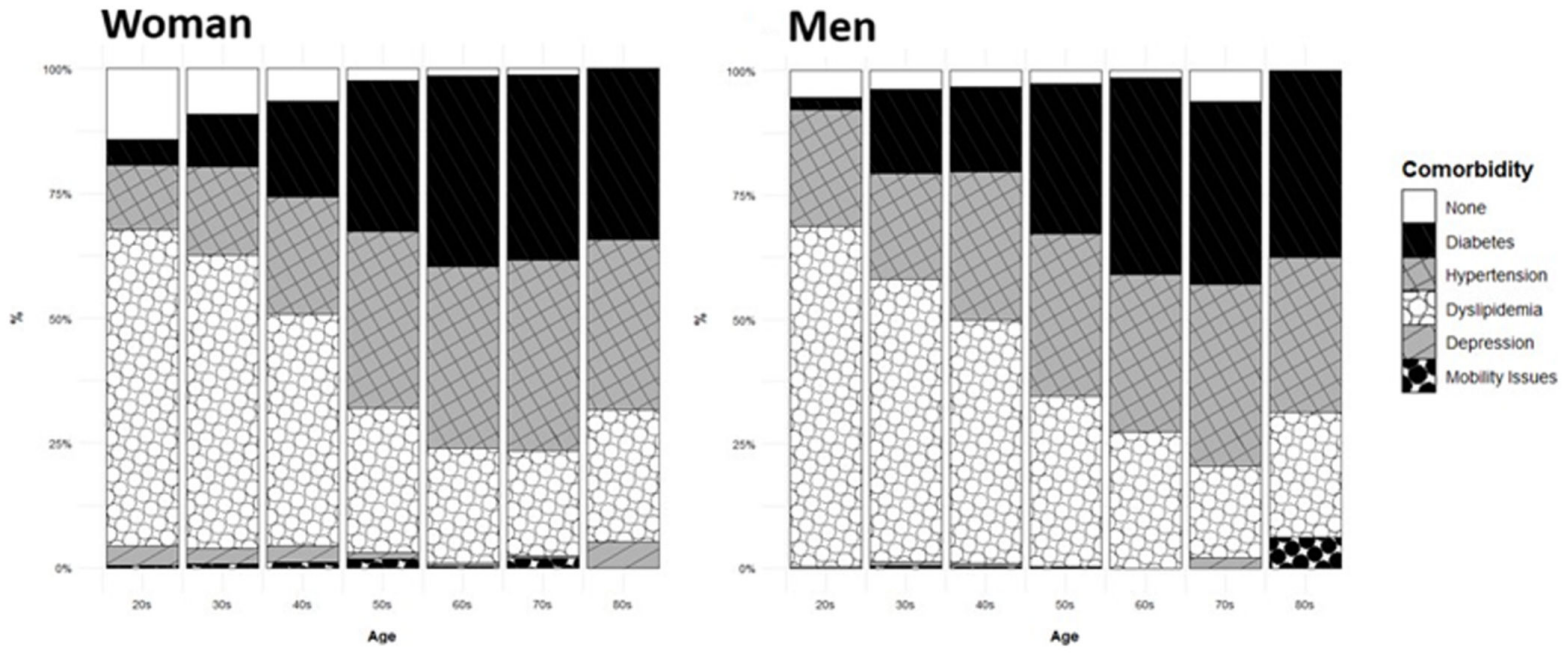
Prevalence and Type of Obesity-Related Comorbidities by Sex and BMI. Left in Men, right in Women. While the prevalence of each comorbidity was statistically different for each intersection in both sexes, they remained comparable in magnitude.

**Figure 3:**
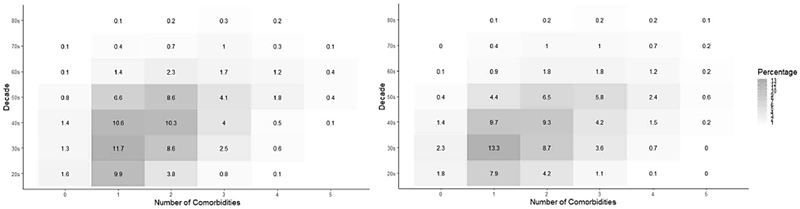
Prevalence and Type of Obesity-Related Comorbidities by Sex and Decade. Left in Men, right in Women. While the prevalence of each comorbidity was statistically different for each intersection in both sexes, they remained comparable in magnitude.

As shown in Table 1, which summarizes the clinical and demographic characteristics by number of simultaneous comorbidities among people with overweight and obesity, the number of co-existing comorbidities increased with both age and BMI. However, as shown in Figure 2, dyslipidemia was especially prevalent among young adults while diabetes, hypertension, and mobility issues markedly increased their prevalence as age increased with diabetes and hypertension peaking in the 70s age group. People without comorbidities were almost non-existent among participants aged 60 years old or older in both sexes as well (Figure 3).

Of those adults with obesity in 2016, 90% met our working definition of NAFLD (FLI > 60) and its prevalence was similar for both sexes at all age groups (Table 2). Conversely, only 0.3% met our working definition of CKD (eGFR ≥ 30 ml/min) in 2016 and 0.09% in 2018. While it was not possible to further characterize this population due to the small number of CKD cases, it is worth highlighting that all patients with CKD were older than 60 years old and had more than 2 or more besides CKD comorbidities (see publicly available code).

**Figure 4:**
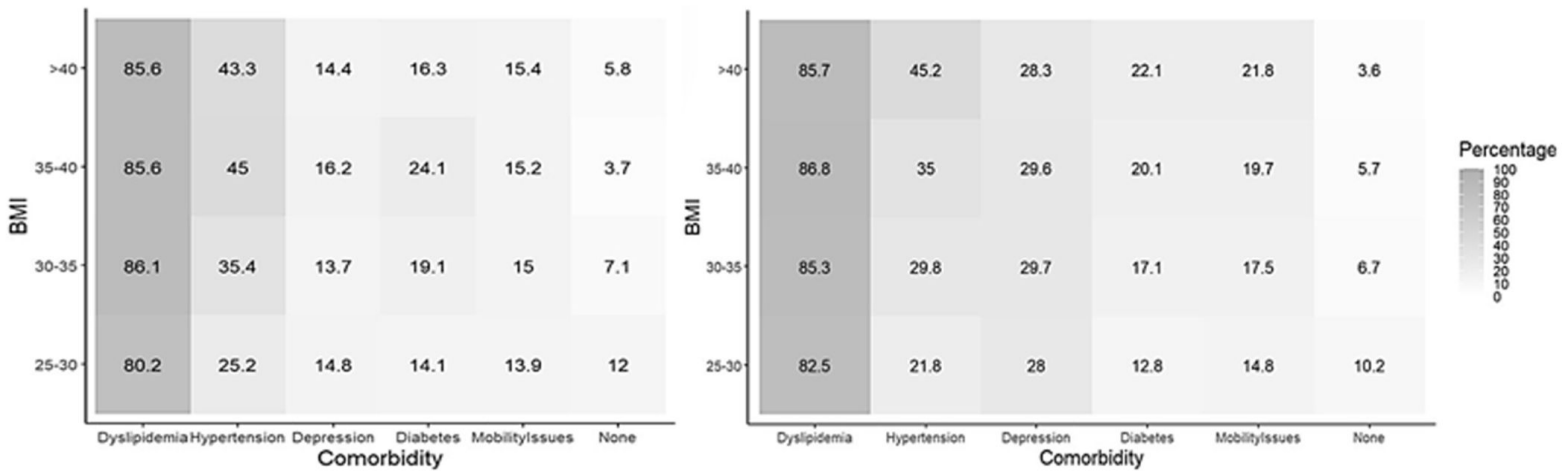
Type of Obesity-Related Comorbidities by Sex and Decade. Left in Men, right in Women. While the prevalence of each comorbidity was statistically different for each intersection in both sexes, they remained comparable in magnitude.

**Figure 5:**
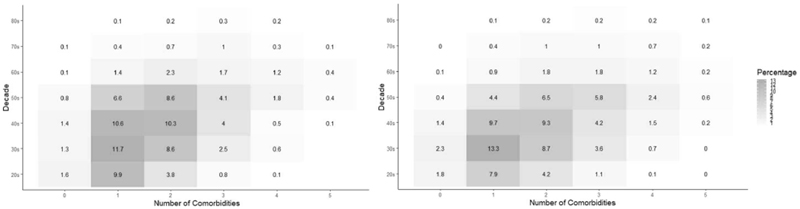
Prevalence and Number of Obesity-Related Comorbidities by Sex and Decade. Left in Men, right in Women. While the prevalence of each comorbidity was statistically different for each intersection in both sexes, they remained comparable in magnitude.

Consistently, the proportional increment in the prevalence of most obesity-related comorbidities to BMI and age (Figures 4 and 5, respectively) was observed for both sexes alike and all comorbidities had their highest prevalence in the older age groups. Additionally, the prevalence of young adults living with 2 or more obesity-related comorbidities decreased during the same period (Supplemental Figure 4) ^19^.

### The heterogeneity of the severity of obesity-related comorbidities (EOSS Phenotypes)

As summarized in Table 3, it was infrequent to see someone living with obesity without at least, subclinical comorbidities (EOSS Stage 1). Moreover, the most prevalent EOSS categories were Stage 2 for the medical dimension (91.9%), and Stage 1 for the functional and mental dimensions (75.1% and 62.8%, respectively). In other words, most people living with obesity have at least one depression or mobility symptom. Consistently, the prevalence of each EOSS category was similar for both sexes (Table 4). Remarkably, the prevalences distribution of EOSS category was similar in individuals with normal weight, overweight and obesity (Supplemental Figures 5 and 6) ^19^.

Finally, as can be seen in Table 5, age accounts for most of the explained variability in the number of obesity-related comorbidities. The addition of BMI, waist-to-height ratio, and sex marginally improved the explained variability, or the root mean square error when they were also included in the model. As summarized in Supplemental Table 1, the mean age of those with normal weight and without comorbidities, decreased in the last two ENSANUT editions.

## Discussion

In this work, we measured the prevalence of obesity-related comorbidities in Mexico across time. Our results describe the heterogeneous clinical expression of obesity in Mexican adults. We documented that the number and type of obesity-related complications have remained largely unchanged between 2006 and 2022. The prevalence of the comorbidities was strongly determined by age and a strong age-dependent divergence in phenotypes was found. For example, all patients with CKD were older than 60 years old while 97% of patients without comorbidities were younger than 60 years old. We observed an increment in the prevalence of people living with obesity without comorbidities, but this phenomenon was limited to young adults.

Since Obesity-related comorbidities are not restricted to metabolic abnormalities the EOSS system^15^considers medical, mental and functional traits ^15^. The most prevalent EOSS categories were Stage 2 for the medical dimension (91.9%), and Stage 1 for the functional and mental dimensions (75.1% and 62.8%, respectively). Our data show that most people living with obesity have at least one depression or mobility symptom, irrespective of the presence or absence of metabolic disturbances.

The stability in the trends in specific obesity-related comorbidities had been previously documented but these have not been considered together and in relation to obesity status^10–12,18,20,21^. However, a novel aspect of this report is the description of an increment in the prevalence of people with obesity without comorbidities (i.e. the metabolically healthy obese phenotype). It is worth noting that the prevalence of people with obesity without comorbidities shrank as older age groups and as more comorbidities are studied (i.e. mental and functional traits). These observations suggest that the metabolically healthy obese phenotype is a specific stage of obesity and still imply relevant health consequences in the long-term.

It is important to highlight that Mexico is a country with one of the largest prevalences of childhood obesity worldwide^1^.

Therefore, the fact that the prevalence of metabolically healthy obese people increased when comparing the last two editions of ENSANUT against the former ones, should be interpreted with caution as it can be explained by a high prevalence of overweight and obese children reaching the cut-off age for our analysis (Supplementary Figure 2) ^19^. Moreover, while similar age and BMI prevalence patterns can be observed in adults with normal weight (Supplemental Figure 6), the mean age of those with normal weight and without comorbidities decreased in recent editions (Supplemental Table 1).

Simultaneously, other factors beyond the shift in the population’s composition may contribute to the increase in the prevalence of metabolically healthy obesity such as increased awareness for screening for obesity-related comorbidities^22,23^. In either case, these results suggest that the prevalence of seemingly infrequent comorbidities such as CKD will increase as the mean age of the population increases especially because age is the strongest predictor of kidney function and major cardiovascular events^24,25^. This is especially important for public policy because even if they are less frequent than other obesity-related comorbidities, major cardiovascular events and end-stage chronic kidney disease impose a larger economic burden^26,27^.

The main strengths of our work include the stratified and nested survey design of ENSANUT^16^, its large sample size when compared with other reports from Latinamerica^28,29^ and the inclusion of non-metabolic comorbidities. Among the limitations of our work, we should mention that our exploration of the psychological and functionality dimensions in EOSS is intrinsically limited as ENSANUT does not inquire about other mental health disorders beyond depression and its mobility questionnaire is not accompanied by physical examination. Consequently, the reported prevalences of emotional and functional obesity-related comorbidities are, most likely, underestimated and the true prevalence of metabolically healthy obesity could be even lower. However, because of how we defined each positive case, we can state that all reported cases correspond to at least, to an EOSS severity level 2 (established disease). Additionally, we should point out that the datasets we analyzed lack data on lack of data on physical activity, smoking, and dietary habits which likely contribute to the remaining unexplained variability in our results.

In summary, the number and type of obesity-related complications have remained unchanged between 2006 and 2022. The prevalence of the comorbidities was strongly determined by age. Consequently, the ongoing aging process of the population will have negative consequences in the Mexican health system in the near future due to the high prevalence of obesity. We observed an increment in the prevalence of people living with obesity without comorbidities, but this phenomenon was limited to young adults. Our results demonstrate the heterogeneous clinical expression of obesity in Mexican adults and provide insight for planning public interventions in the coming years and provide insight for future epidemiological and therapeutical research studies.

## Supplementary Material

Supplementary

## Figures and Tables

**Table 1:** Clinical Characteristics of the Studied Population by Number of Comorbidities Among People With Overweight and Obesity. Unless otherwise stated, data are means ± standard deviation for continuous variables or the 95% confidence interval for the prevalence of categorical variables. n=14 844, corresponding to the population with a BMI above 24.9.

	Number of Comorbidities											
	0		1		2		3		4		5	
**n**	1298		6194		4424		2079		699		150	
**Age**	35.8	±11.0	38.8	±11.9	44.5	±13.4	50.38	±13.8	55.66	±13.0	60.54	±13.1
**Women (n, %)**	780.0	60.1%	3877.0	62.6%	2858	64.6%	1425	68.5%	519	74.2%	113	75.3%
**BMI**	29.2	±3.6	30.1	±3.9	30.85	±4.2	31.28	±4.4	31.69	±4.3	31.45	±3.9
**Total Cholesterol**	164.1	±22.6	184.8	±49.6	191.14	±53.3	192.18	±54.5	194.82	±52.5	192.96	±60.6
**LDL**	97.7	±21.0	112.7	±40.5	118.14	±44.6	118.58	±44.4	121.34	±43.5	119.05	±49.8
**HDL**	46.5	±7.8	41.0	±10.7	40.85	±11.1	40.3	±11.0	40.45	±11.8	39.1	±10.8
**Triglycerides**	101.7	±26.9	161.2	±97.5	165.34	±93.2	171.05	±97.7	169.85	±89.1	177.45	±85.9
**Fasting Glucose**	89.3	±10.5	92.5	±15.4	108	±46.3	129.83	±67.8	155.39	±80.0	169.83	±84.9
**Systolic Blood Pressure**	114.5	±12.0	117.2	±12.6	125.13	±18.5	131.11	±20.4	137.58	±22.1	138.91	±21.2
**Diastolic Blood Pressure**	73.4	±9.40	75.8	±9.3	80.16	±12.5	82.56	±13.0	84.57	±12.8	82.19	±11.9
**Trig/HDL**	2.3	±0.70	4.2	±3.1	4.35	±3.1	4.55	±3.2	4.45	±2.6	4.74	±2.5
**Hypertension (n, %)**	Does not apply		287	4.6%	1694	38.3%	1430	68.8%	622	89.0%	Does not apply	
**Diabetes (n, %)**	Does not apply		72	1.2%	772	17.5%	879	42.3%	481	68.8%	Does not apply	
**Dyslipidaemia (n, %)**	Does not apply		5405	87.3%	4160	94%	2003	96.3%	686	98.1%	Does not apply	
**Depression (n, %)**	Does not apply		295	4.8%	1442	32.6%	1124	54.1%	519	74.2%	Does not apply	
**Impaired Mobility (n, %)**	Does not apply		135	2.2%	780	17.6%	801	38.5%	488	69.8%	Does not apply	

**Table 2:** Prevalence of NAFLD Among People with Obesity. NAFLD was defined as FLI ≥ 60. Percentages were calculated vs the total of people meeting each cell criteria as denominator.

Non-Alcoholic Fatty Liver Disease (2016)		
	Men (27%)	Women (73%)
20 to 29 years old	100 %	69.4 %
30 to 39 years old	100 %	85.6 %
40 to 49 years old	98.8 %	88.2 %
50 to 59 years old	98.4 %	92.5 %
60 to 69 years old	98.0 %	93.4 %
≥ 70 years old	100 %	86.8 %

**Table 3:** Prevalence of each EOSS subtype. Percentages do not add up to 100% as there can be overlap between definitions and between comorbidities within each stage. n=14844.

	Stage 0	Stage 1	Stage 2	Stage ≥ 3
**Medical**	4.2%	3.9%	91.9%	69.9%
**Mental**	27.7%	62.8%	25.3%	NA
**Functional**	38.3%	75.1%	17.8%	NA

**Table 4:** Prevalence of EOSS categories by sex. Percentages do not add up to 100% as there can be overlap between definitions and between comorbidities within each stage

Women (n = 9572)					Men (n = 5272)			
Severity	Stage 0	Stage 1	Stage 2	Stage ≥ 3	Stage 0	Stage 1	Stage 2	Stage ≥ 3
**Medical**	5.4%	4.2 %	90.4 %	29.4 %	3.0 %	5.2 %	91.8 %	40.6 %
**Mental**	26.8 %	61.9 %	29.6 %	NA	29.7 %	65.1 %	14.4 %	NA
**Functional**	38.2 %	75.3 %	18.5 %	NA	38.5 %	74.8 %	15.1 %	NA

**Table 5:** Analysis of Age’s Contribution to the Explained Variability o f the Number of Obesity-Related Comorbidities. Explained variability in the number of obesity-associated comorbidities by different multivariable linear models. WHtR= Waist circumference to Height Ratio. RMSE = Root Mean Square Error.

Linear Model	RMSE	R^2^
Number of comorbidities ~ Age	0.95	16%
Number of comorbidities ~ Age + Sex	0.95	17%
Number of comorbidities ~ Age + Sex + BMI	0.94	19%
Number of comorbidities ~ Age + Sex + BMI +WHtR	0.94	19%

## Data Availability

Data is available at: https://ensanut.insp.mx/encuestas/ensanutcontinua2021/descargas.php.

## Esearch In Context

### Evidence before this study

Obesity is highly prevalent in Mexico and Latin America. However, its impact on mobility, mental health, or the prevalence of metabolically healthy obesity has been longitudinally studied for this region.

### Added value of this study

This study uses measurement data from multiple national population surveys to estimate trends in obesity comorbidities and phenotypes. It is the first example of comorbidity patterns of obesity in LA in middle-income countries where obesity is highly prevalent.

### Implications of all the available evidence

The rising prevalence of obesity and the ageing of the population will cause additional burden to the Mexican population and health system.
