# Supplementary material for: Clinical Heterogeneity and Transitions of Obesity in Mexico. A Longitudinal Analysis of Multiple Representative National Surveys
Source: J Clin Endocrinol Metab. Author manuscript; Available in PMC 2025 Apr 4. (PMC7617558; doi:10.1210/clinem/dgaf158)
Supplement: Supplementary [file EMS203654-supplement-Supplementary.pdf]

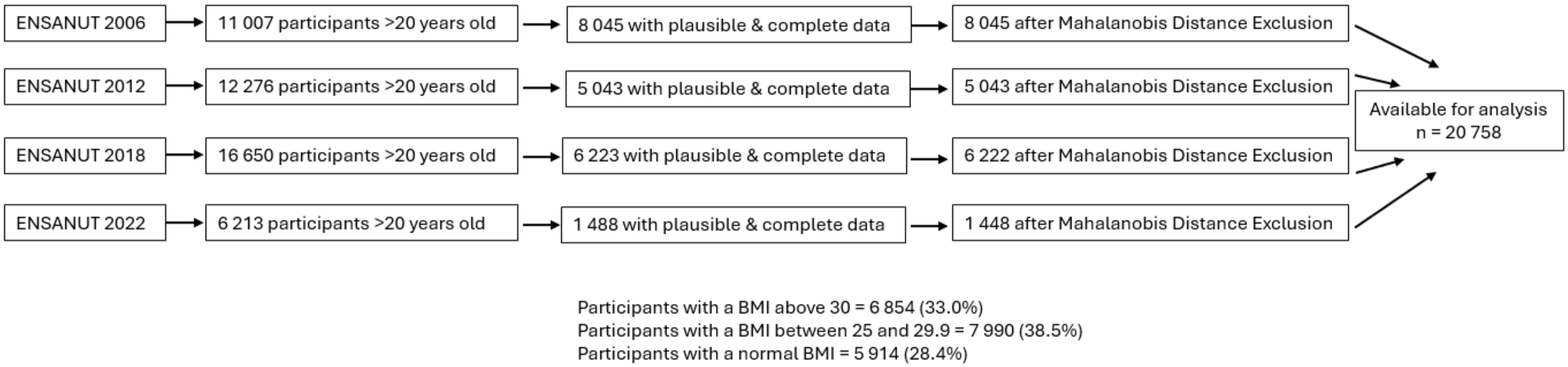

Supplemental Figure 1. Data Cleaning Flowchart.

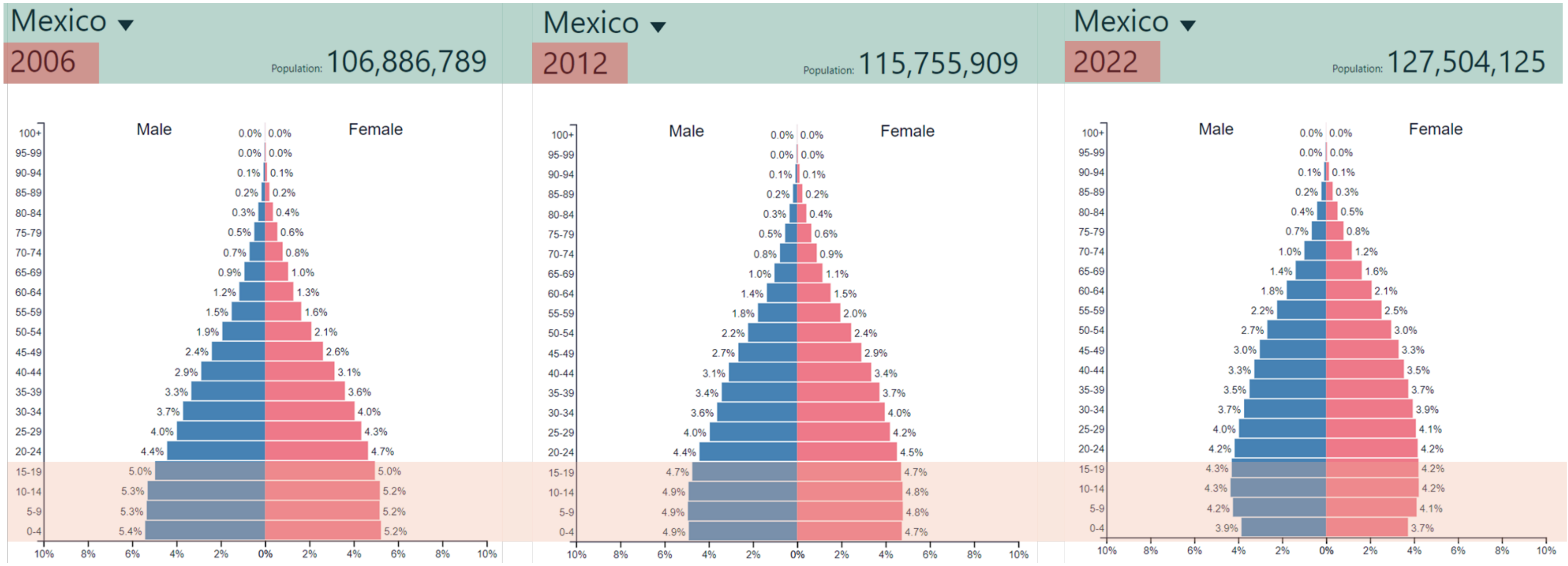

Supplemental Figure 2. Change in Mexican Population Age Composition During the Studied Years.

Highlighted in orange, the change in the proportion of paediatric population during the relevant timeframe.

Data accessed on October 2024 at: https://www.populationpyramid.net/es/m%C3%A9xico/

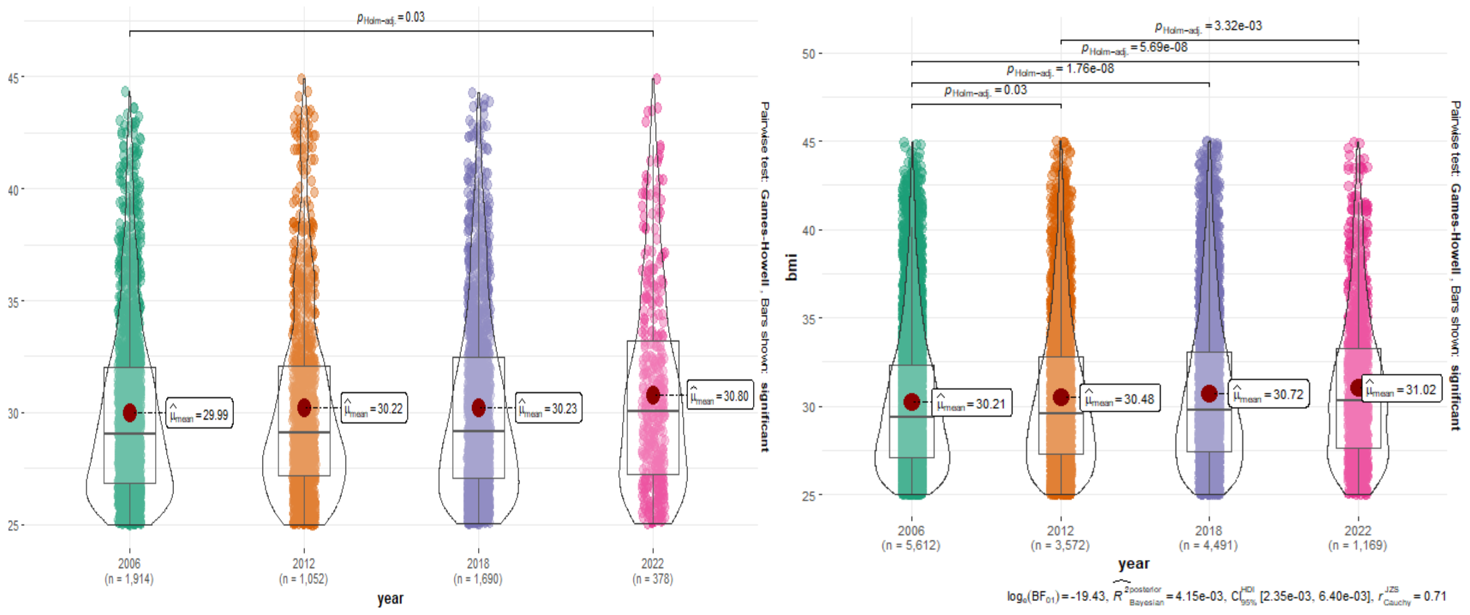

Supplemental Figure 3. A) BMI Among Young Adults During the Studied Years. B) BMI Among All Ages Across Time

The cut-off age for young adults was 36 years old as the operative definition for adult in ENSANUT is 20 years old and our timeframe encompasses.

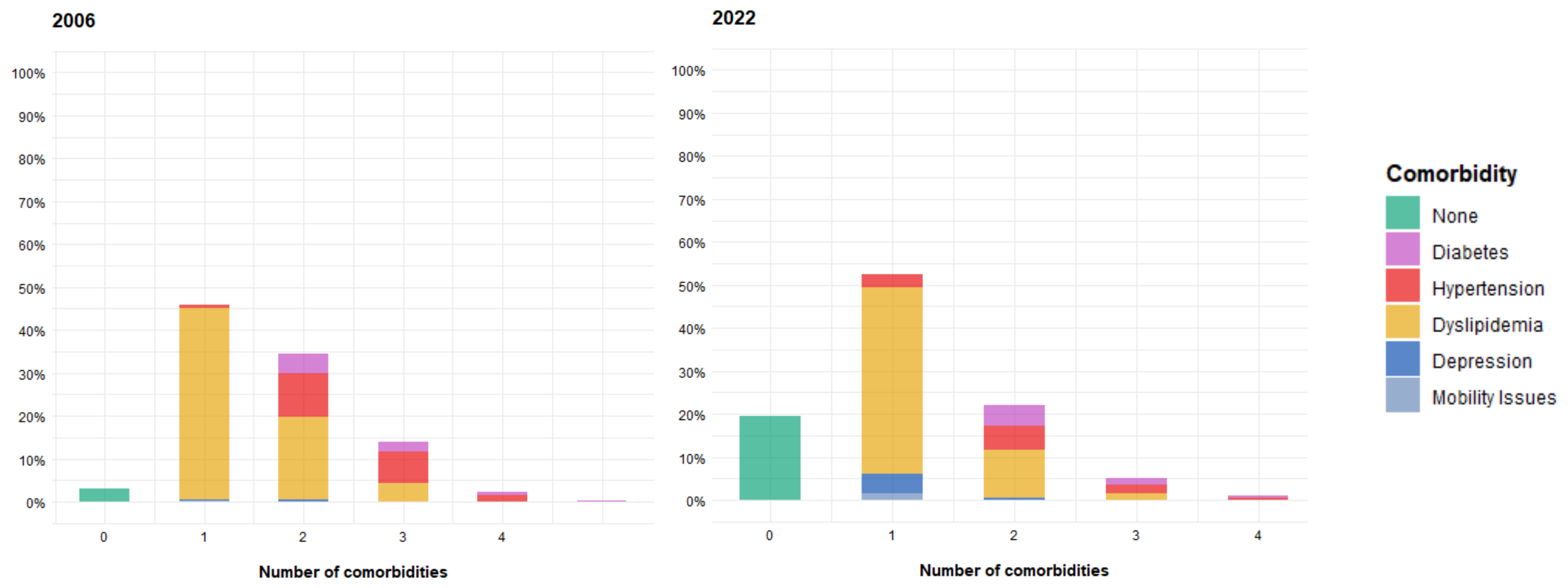

Supplemental Figure 4. Obesity-related comorbidities among those younger than 20 years old in 2006 and 2022.

For 2006 n = 1932. For 2022 n = 384.

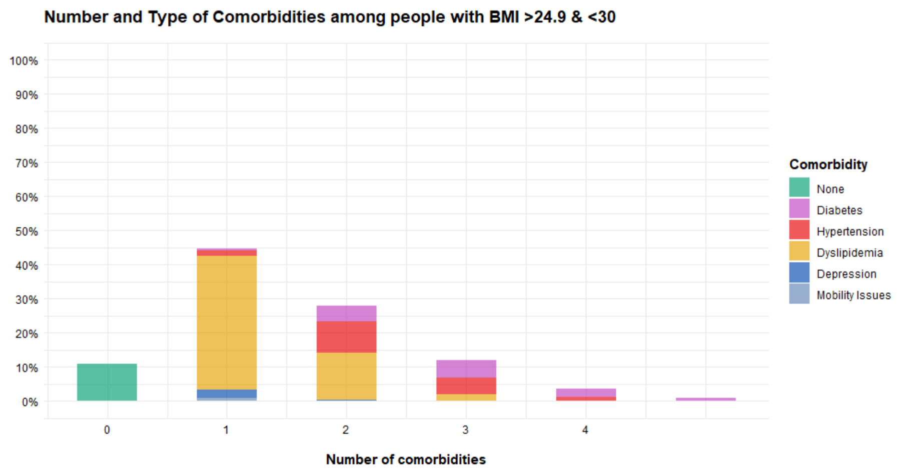

BMI >25 & <30				
	Stage 0	Stage 1	Stage 2	Stage ≥ 3
Medical	8.5%	6.0%	85.5%	26.0%
Mental	30.4%	61.5%	22.6%	NA
Functional	37.0%	75.0%	14.5%	NA

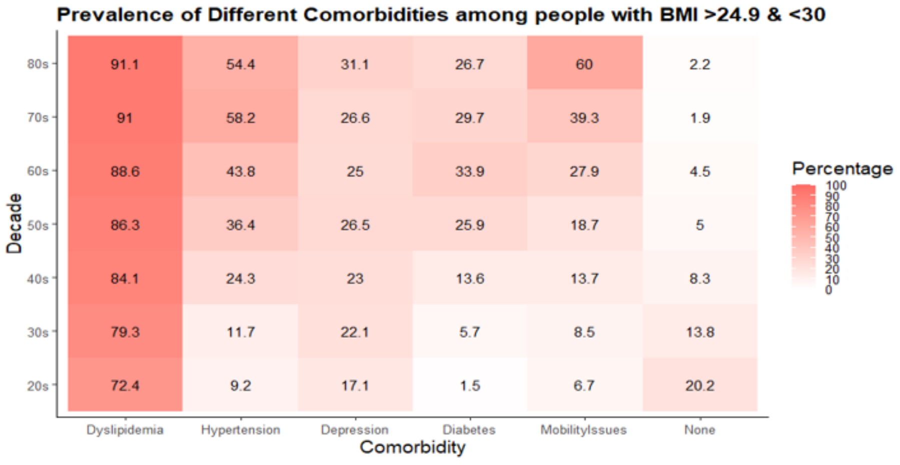

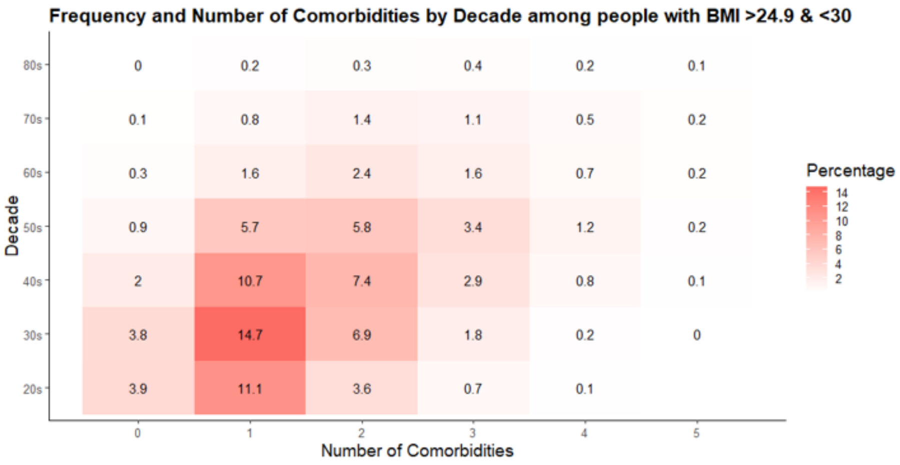

Supplemental Figure 5. Prevalence of EOSS categories among those with a BMI between 25 and 30 kg/m^2.

	2006<br>n=2103	2012<br>n=1240	2018<br>n=1437	2022<br>n=240
**Diabetes (%)**	11.5	9.2	9.5	9.8
**Dyslipidaemia (%)**	92.3	67.3	53.7	52.9
**Depression (%)**	24.8	19.4	18.5	14.2
**Mobility Impairment (%)**	19.2	7.1	8.2	12.5
**Hypertension (%)**	17.8	19.0	13.6	12.5
	**Mean age of those with normal weight and no comorbidities**			
**Years (St.Dev.)**	41.7 (± 17.8)	43.8 (± 19.1)	35.3 (± 11.6)	36.1 (± 11.5)

Supplemental Table 1. Prevalence of metabolic comorbidities among individuals with normal weight across ENSANUT editions.
